# The diagnostic value of imaging techniques for keratoacanthoma: A review

**DOI:** 10.1097/MD.0000000000032097

**Published:** 2022-12-30

**Authors:** Xiujuan Zhang, Jiahong Shi, Zhixia Sun, Ting Dai

**Affiliations:** a Department of Ultrasound, China-Japan Union Hospital of Jilin University, Changchun, China.

**Keywords:** clinical diagnosis, differential diagnosis, imaging examination, keratoacanthoma, skin tumor

## Abstract

Keratoacanthoma (KA) is a fast-growing skin tumor with solitary KA being the most common type. KAs rarely metastasize and subside spontaneously. Although histopathology is the gold standard for the diagnosis of KA, its histopathological features are sometimes difficult to distinguish from those of other skin tumors. Imaging studies have certain advantages in the preoperative diagnosis of KA; they not only show the exact shape of the lesion but can also accurately determine the extent of the lesion. Combined with histopathological examination, these findings help establish a diagnosis. By summarizing the imaging features of KA, this article aimed to improve radiologists’ understanding of the disease and help in the clinical and differential diagnosis of KA.

## 1. Introduction

Keratoacanthoma (KA) is a fast-growing skin tumor that usually occurs in the sun-exposed parts of the skin. It is more commonly seen in middle-aged and elderly patients, and its incidence rate is higher in men than in women. The specific etiology and pathogenesis of this disease are unknown. Clinically, they are divided into single, multiple, and eruptive types. Among them, single-type tumors are the most common and rarely metastasize.^[1]^ In addition, research has shown that the disease can subside spontaneously; therefore, typical KA generally undergoes 3 clinical stages: proliferation, stabilization, and regression.^[2]^ Although the disease can resolve spontaneously, its tendency to recur and undergo malignant transformation means that the corresponding treatment is performed as clinically indicated. Surgery is the main treatment for single-type KA; however, for patients who cannot be surgically treated, methotrexate^[3]^ and interferon^[4]^ can be injected into the lesion, and tretinoin can be used for multiple-type KA and other rashes. Moreover, the histopathological features of KA are sometimes difficult to distinguish from those of other skin tumors, particularly squamous cell carcinoma (SCC),^[5,6]^ which may lead to unnecessary and excessive treatment. At present, imaging techniques include ultrasound (US), computed tomography (CT), magnetic resonance imaging (MRI), and positron emission tomography/computed tomography (PET/CT). This review summarizes the aforementioned imaging manifestations of KA and is designed to improve the understanding of doctors regarding the role of imaging in this disease, thus contributing to the clinical diagnosis and differential diagnosis of KA.

## 2. Methods

**Figure 1.**
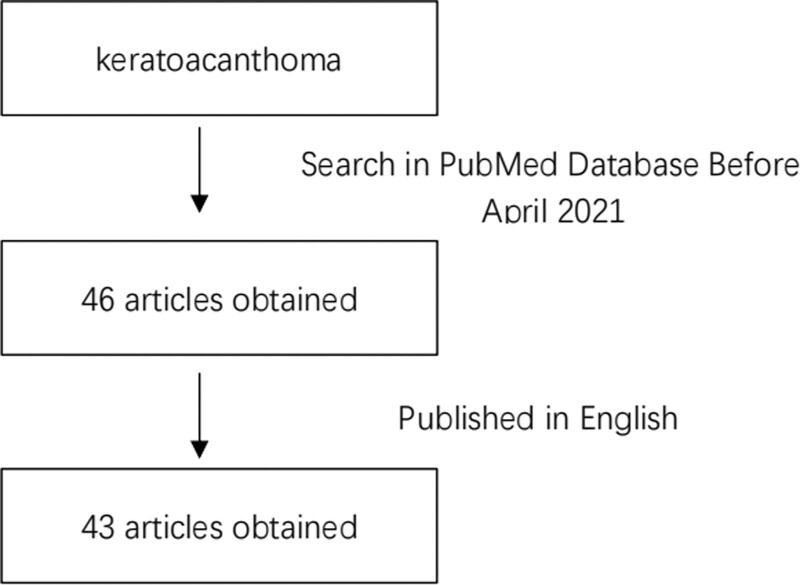
Method of literature selection. Keratoacanthoma was used as the keyword to search the PubMed database, and the identified articles were combined with one of the following keywords in a separate search: ultrasonography, CT, positron emission tomography, and MRI. CT = computed tomography, MRI = magnetic resonance imaging.

Relevant articles were retrieved using the following search strategies: “keratoacanthoma” was used as the keyword to search in the PubMed database, and the identified articles were combined with one of the following keywords in a separate search: ultrasonography, CT, PET/CT, and MRI. The inclusion criterion was articles published before April 2021, and the exclusion criterion was non-English-language papers. Three of the 46 articles found in the final search were excluded because they were not in English language. An overview of the aforementioned search strategy is presented in Figure 1. The patient information and images were approved by the ethics committee of our hospital. The inclusion criteria were as follows: postoperative pathological diagnosis of KA; preoperative imaging studies (US, CT, PET/CT, or MRI); and patients treated in our hospital from January 2012 to April 2021. The exclusion criterion was Muir-Torre syndrome, which is characterized by KA. Two doctors independently screened cases based on the inclusion and exclusion criteria.

## 3. Results

### 3.1. Sonographic features of KA

US is increasingly used for the early detection of skin tumors because of its safety, non-invasiveness, and convenience.^[7,8]^ The US manifestations of KA are as follows.

**Figure 2.**
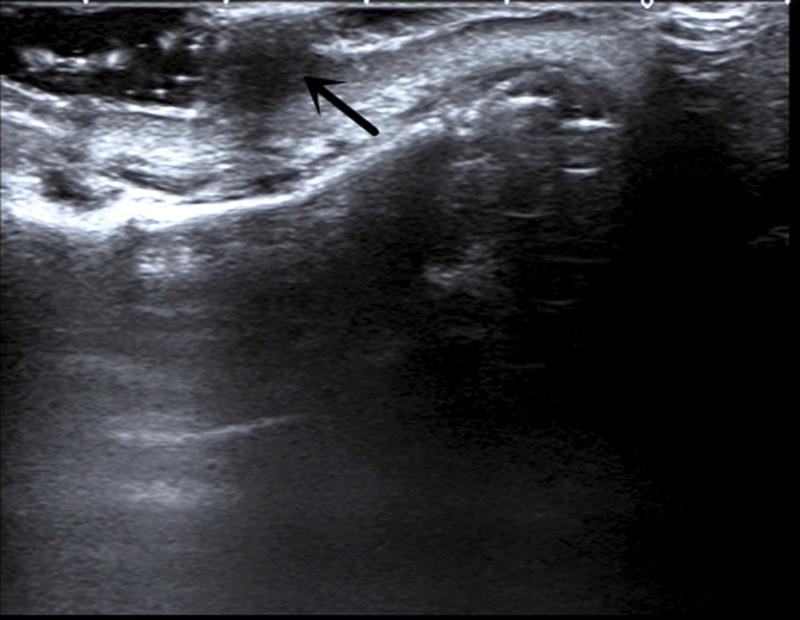
Two-dimensional US shows hypoechoic mass with uniform echo within the skin layer with clear boundaries (black arrow). US = ultrasound.

**Figure 3.**
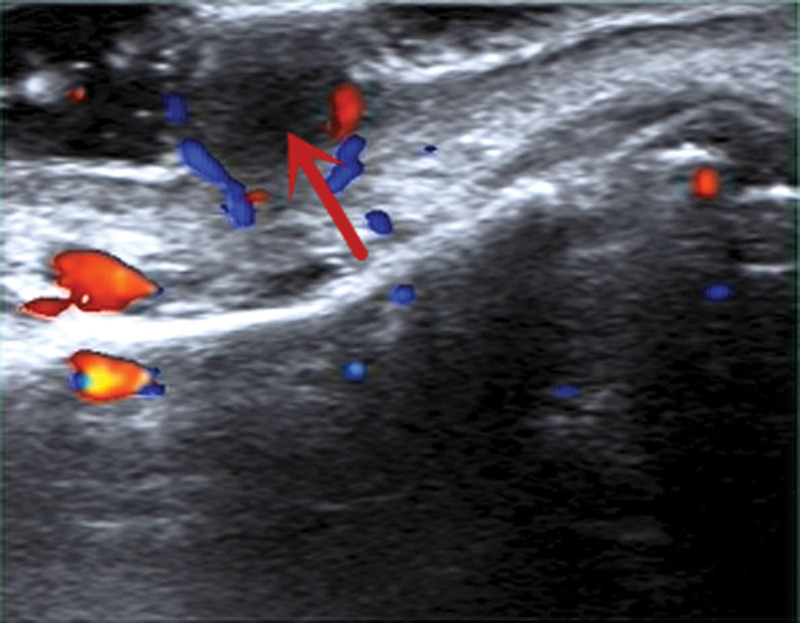
CDFI shows hypoechoic mass with lack of blood supply (red arrow). CDFI = color doppler flow imaging.

**Figure 4.**
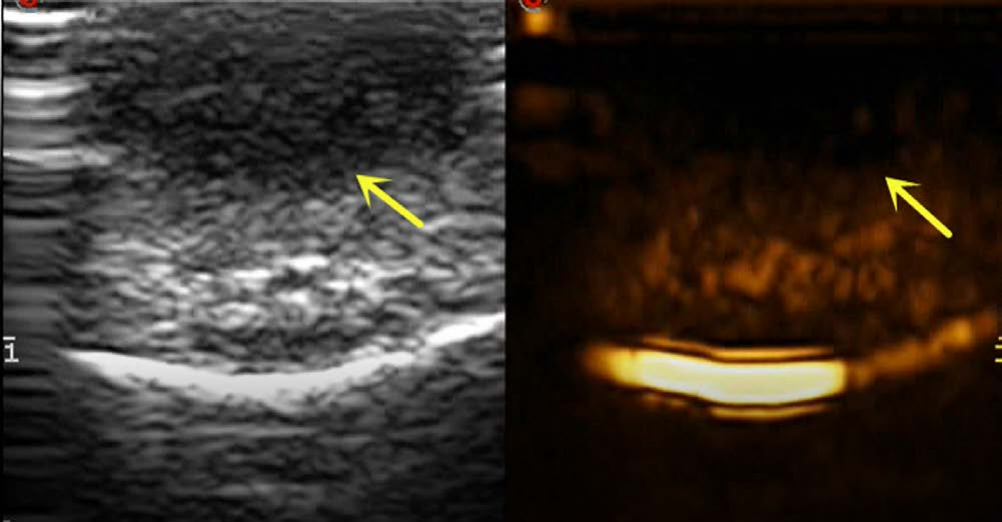
CEUS shows mass is low enhancement (yellow arrow). CEUS = contrast-enhanced ultrasound.

1. Two-dimensional US features: hypoechoic masses with uniform echo within the skin layer, with clear boundaries and regular morphology (Fig. 2).
2. Color Doppler flow imaging (CDFI) characteristics: The blood supply in the tumor is closely related to its clinical stage. In the proliferation phase, CDFI shows blood enrichment, whereas in the regression phase, there is a lack of blood supply^[9]^ (Fig. 3).
3. Contrast-enhanced ultrasound (CEUS) characteristics: There is a dearth of literature regarding CEUS. In our hospital, the performance of CEUS is slow forward with rapid regression and low enhancement (Fig. 4).

### 3.2. CT features of KA

**Figure 5.**
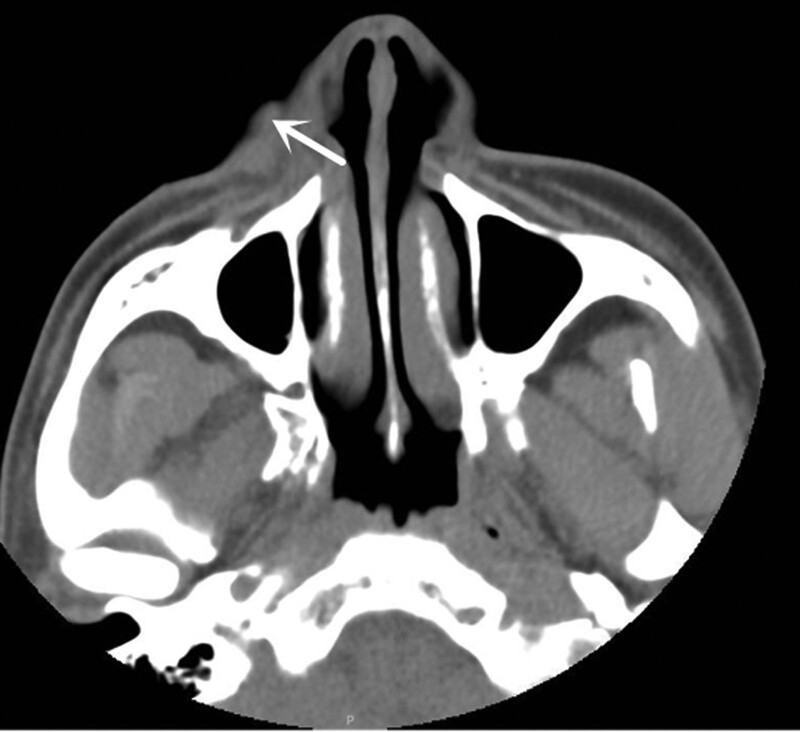
CT shows exogenous, dome-shaped nodule mass with uniform and low density with skin clear boundary (white arrow). CT = computed tomography.

At present, CT is relatively popular and can clearly show the range of lesion sizes in skin tumors. KA has no specific features on CT imaging; it appears as an exogenous, dome-shaped, or cup-shaped skin nodule with clear boundaries, is relatively uniform and has low density (Fig. 5), has rare or almost no invasive growth, and displays mild enhancement.^[10]^

### 3.3. MRI features of KA

The advantage of MRI is not only that it has higher soft tissue resolution, but also that it can perform cross-sectional imaging in any orientation. KA lacks in specific MRI features. The T1-weighted image of the lesion demonstrates an intermediate signal, and the T2-weighted image demonstrates a mixture of intermediate and high signals with enhancement of the surrounding thin edges.^[10–12]^

### 3.4. PET/CT features of KA

PET/CT is currently the most advanced molecular imaging technology and is widely used in diagnosis, differential diagnosis, and efficacy evaluation.^[13,14]^ However, because of its high cost, patients with KA do not usually undergo PET/CT examination. Nonetheless, when evaluating PET/CT images, radiologists should pay attention to the differentiation of benign from proliferative diseases, such as the proliferative stage of KA, to avoid misdiagnosis.^[15]^

### 3.5. Differential diagnosis of KA

KA is significantly different from SCC and basal cell carcinoma (BCC) in terms of treatment and prognosis; therefore, differential diagnosis is crucial.

#### 3.5.1. SCC.

Because of the histopathological similarities between KA and SCC, KA is often misdiagnosed as SCC, leading to unnecessary overtreatment. However, differences in imaging characteristics can help distinguish between them. On US, most SCC are aggressive and often involve the dermis and subcutaneous skin layers.^[16]^ The masses were mostly heterogeneous and hypoechoic with unclear borders, irregular shapes, very rich blood supply, and lymph node metastasis. KA is generally confined to the skin layer, with a clear boundary of the tumor mass, and rarely metastasizes. On CT, SCC appears as a soft tissue mass with a relatively uniform density but with aggressive growth that invades adjacent structures. This is a moderate or significant enhancement.^[10]^ KA usually does not invade surrounding structures and is only slightly enhanced. On MRI, most skin SCC manifest as low to intermediate signals on T1-weighted images and slightly high to high signals on T2-weighted images. The tumor may show uneven enhancement.^[11]^ KA generally shows a peripheral thin rim enhancement. Therefore, although it may be difficult to distinguish between them based on histopathology, imaging technology may aid in the clinician’s diagnosis.

#### 3.5.2. BCC.

BCC is a relatively common skin cancer that also tends to occur in sun-exposed parts of the skin.^[17]^ It is sometimes difficult to distinguish KA from BCC. On US, BCC is a solid hypoechoic or non-echoic mass with irregular borders and a hyperechoic spot. These hyperechoic spots can help distinguish BCC from other types of skin tumors.^[18]^ KA generally has a clear boundary and a regular shape. On CT, BCC has equal or low density relative to the adjacent muscle tissue. On MRI, BCC shows intermediate to high signal intensity on T1-weighted images and high signal intensity on T2-weighted images. BCC grows slowly, which helps to distinguish it from KA. However, it is difficult to differentiate between them using imaging studies, and the diagnosis ultimately depends on histopathological examination.

## 4. Discussion

Few studies have focused on imaging in KA, and most are related to direct immunofluorescence of skin biopsy or histopathological diagnosis. Therefore, radiologists lack a systematic understanding of the imaging features of KA. This article reviews the characteristics of several different imaging examinations of KA, which can be summarized as follows: 2-dimensional US: morphologically regular hypoechoic mass with a clear inner boundary of the skin layer; CT: exogenous, dome-shaped mass or cup-shaped skin nodule with clear boundaries, relatively uniform, low density, rare or almost no invasive growth, and displaying mild enhancement; and MRI: intermediate signal on T1-weighted images, mixed intermediate and high signal on T2-weighted images, and peripheral thin rim enhancement. In addition, studies have shown that KA at the proliferative stage has a clear tendency for vascularization. CDFI and CEUS can capture changes in blood flow to the lesion, thereby effectively identifying the clinical stage of the tumor. Ruiz-Villaverde^[9]^ reported the US and histopathological findings of hospitalized patients and found that CDFI showed a rich blood supply if the tumors were in the proliferative phase and a lack of blood supply if they were in the regression phase. Meanwhile, the CDFI of KA cases from our hospital demonstrated decreased blood supply to the lesion, and CEUS showed low enhancement, suggesting that the cases were in the regression phase. However, there is a paucity of literature on CDFI and CEUS of KA, and much research is still needed to describe their features.

Because KA lacks specific characteristics in terms of clinical features and histopathological analysis, it is sometimes difficult to distinguish it from SCC and BCC. Most KAs can be cured by treatment and rarely metastasize. Poorly differentiated SCC is highly aggressive and prone to metastasis and can metastasize to multiple organs in the late stage, which is difficult to treat and has a poor prognosis.^[19]^ BCC is locally invasive, can damage surrounding tissue, and has a high recurrence rate.^[20]^ KA and other skin tumors are significantly different in terms of treatment and prognosis; therefore, distinguishing between them is very important. Imaging examination can not only show the exact shape, scope, and extent of the lesion but also identify subclinical and recurring lesions. Therefore, it is necessary to understand the imaging characteristics of these diseases and recognize the differences between them and the imaging findings in KA. US is the preferred imaging modality for KA because it is inexpensive, convenient, and noninvasive. Compared to CT, MRI has superior resolution for soft tissue and certain advantages for the qualitative diagnosis of KA. In addition, KAs at the proliferative stage have an apparent tendency for vascularization. CDFI and CEUS can reveal changes in blood flow to the lesion, thereby effectively identifying the clinical stage of the tumor. The degree of neovascularization is variable, and it is important to complement surgical treatment with drugs such as intralesional methotrexate injection. Although the gold standard for skin tumors is histopathological examination, pathological specimens may sometimes lead to an inconclusive diagnosis. Furthermore, owing to the small sampling range, histopathological examination sometimes fails to clarify the relationship between the lesion and the surrounding tissue. Therefore, it can be concluded that the diagnosis of KA by imaging not only improves radiologists’ understanding of the disease but also better guides its diagnosis and treatment by clinicians. At present, whether KA is a benign or malignant tumor remains unclear^[1]^; therefore, caution must be exercised in its clinical treatment and patient follow-up is crucial.

In conclusion, KA is a fast-growing skin tumor. As most patients undergo direct immunofluorescence of skin biopsy and are diagnosed by histopathology, few imaging examinations have been conducted. US shows a hypoechoic mass and uniform echo within the skin layer, with a clear boundary and regular morphology. CT demonstrated a low-density nodule with a clear boundary and a relatively uniform distribution. T1-weighted MRI images show an intermediate signal, while T2-weighted images show mixed intermediate and high signals with peripheral thin rim enhancement.

This systematic review of imaging features may help in the differential diagnosis of KA and improve radiologists’ understanding of the disease to better guide clinical diagnosis and treatment.

## Author contributions

**Conceptualization:** Xiujuan Zhang, Jiahong Shi, Zhixia Sun, Ting Dai.

**Data curation:** Xiujuan Zhang, Jiahong Shi, Zhixia Sun, Ting Dai.

**Formal analysis:** Xiujuan Zhang, Jiahong Shi, Zhixia Sun, Ting Dai.

**Investigation:** Xiujuan Zhang, Jiahong Shi, Ting Dai.

**Writing – original draft:** Xiujuan Zhang, Jiahong Shi, Zhixia Sun.
